# The *In Vivo* Antidiabetic Activity of *Nigella sativa* Is Mediated through Activation of the AMPK Pathway and Increased Muscle Glut4 Content

**DOI:** 10.1155/2011/538671

**Published:** 2011-04-14

**Authors:** Ali Benhaddou-Andaloussi, Louis Martineau, Tri Vuong, Bouchra Meddah, Padma Madiraju, Abdellatif Settaf, Pierre S. Haddad

**Affiliations:** ^1^Department of Pharmacology and Montreal Diabetes Research Center, Université de Montréal, Montréal, QC, Canada H3C 3J7; ^2^Institut des Nutraceutiques et des Aliments Fonctionnels, Laval University, Quebec City, QC, Canada G1K7P4; ^3^Research Team in Pharmacokinetics, Laboratory of Pharmacology and Toxicology, Faculty of Medicine and Pharmacy, Mohammed V University, Rabat-Souissi, Morocco

## Abstract

The antidiabetic effect of *N. sativa* seed ethanol extract (NSE) was assessed in *Meriones shawi* after development of diabetes. *Meriones shawi* were divided randomly into four groups: normal control, diabetic control, diabetic treated with NSE (2 g eq plant/kg) or with metformin (300 mg/kg) positive control, both administered by daily intragastric gavage for 4 weeks. Glycaemia and body weight were evaluated weekly. At study's end, an Oral Glucose Tolerance Test (OGTT) was performed to estimate insulin sensitivity. Upon sacrifice, plasma lipid profile, insulin, leptin, and adiponectin levels were assessed. ACC phosphorylation and Glut4 protein content were determined in liver and skeletal muscle. NSE animals showed a progressive normalization of glycaemia, albeit slower than that of metformin controls. Moreover, NSE increased insulinemia and HDL-cholesterol, compared to diabetic controls. Leptin and adiponectin were unchanged. NSE treatment decreased OGTT and tended to decrease liver and muscle triglyceride content. NSE stimulated muscle and liver ACC phosphorylation and increased muscle Glut4. These results confirm NSE's previously reported hypoglycaemic and hypolipidemic activity. More significantly, our data demonstrate that *in vivo* treatment with NSE exerts an insulin-sensitizing action by enhancing ACC phosphorylation, a major component of the insulin-independent AMPK signaling pathway, and by enhancing muscle Glut4 expression.

## 1. Introduction

Diabetes is a chronic disease that occurs when the pancreas does not produce enough insulin, and/or when the body cannot effectively use the insulin it produces. Hyperglycaemia, or high blood sugar, is a common effect of uncontrolled diabetes and over time leads to serious damage to many of the body's systems, especially the nerves, kidney, and blood vessels [1]. The World Health Organization (WHO) estimates that more than 180 million people worldwide have diabetes. This number is likely to more than double by 2030 [1]. Therapeutic interventions for patients with type II diabetes include diet, exercise, oral hypoglycemic agents, and/or insulin. For centuries or even millennia, medicinal plants around the world have also been used to treat the disease; over a thousand plants being reported to combat diabetes or its major symptoms [2–4]. The hypoglycaemic action of these plants is exerted by several mechanisms, such as stimulation of insulin production [5], enhancement of insulin sensitivity [6], or inhibition of intestinal amylase [4].

*N. sativa* is a herbaceous plant growing to about 20–30 cm in height, commonly known as black seed because of the small triangular black seeds it generates. The plant is also known as Blessed Seed (Arab: Habbat ul Baraka, or Habbat ul Sauda). It has been consumed for more than 2000 years, is used extensively in the traditional medicine of many southern Mediterranean and Middle Eastern countries, and has been shown to produce multisystemic beneficial actions [7], including hypocholesterolemic [8], antioxidant [9, 10], and anti-inflammatory effects [11].

The hypoglycaemic and antidiabetic effect of *N. sativa* has been reported by numerous *in vivo* and *in vitro* scientific studies [9, 12–25]. In a recent study, we have demonstrated that *N. sativa* seed ethanol extract (NSE) exhibits the remarkable ability *in vitro* to concomitantly increase insulin secretion, induce proliferation of pancreatic *β* cells, and stimulate glucose uptake in skeletal muscle and fat cells [24]. On the other hand, most of the *in vivo* studies of the antidiabetic effect of *N. sativa* were carried out on models of type I diabetes. We therefore investigated the effects of NSE on the diabetic *Meriones shawi* that represents a model of type II diabetes associated with hyperinsulinemia and dyslipidemia. We have also attempted to determine some of the mechanisms of action through which NSE may exert its antidiabetic effect, notably adipokines, AMP-kinase (AMPK) dependent signalling, and Glut4 protein content.

## 2. Material and Methods

### 2.1. Reagents and Antibodies

Antibodies against pan-specific and phosphorylated acetyl CoA carboxylase (ACC) (Ser 79), as well as Glut4 antibodies were purchased from Cell Signaling Technology (Danvers, MA, USA). Secondary HRP-conjugated antibodies were purchased from Jackson Immunoresearch (Cedarlane Laboratories, Hornby, ON). Protein assay kit was purchased from Pierce (Brockville, ON). Rat Insulin-specific RIA kit, Rat Leptin RIA kit, and Mouse Adiponectin RIA kit-125T were purchased from Linco Research Inc (Saint Charles, MO). Sodium pentobarbital, triglyceride, and free glycerol reagents as well as D-glucose were purchased from Sigma-Aldrich (Saint Louis, MO).

### 2.2. Plant Material

Seeds of *N. sativa* were obtained from an herbalist in Rabat, Morocco in August 2005 and were authenticated by an experienced botanist (Professor A. Oulyahya, Institut Scientifique, Rabat, Morocco). A voucher specimen has been deposited in the herbarium of the Institut Scientifique of Rabat (no. 10359). Seeds were washed, dried, and then powdered with an electric microniser. Powder was extracted three times with 80% ethanol and the solvent was evaporated at 40°C under reduced pressure. This procedure resulted in a two-phased extract. The oily and the solid phases were recombined in proportion to their yield (typically 70% and 30%, resp.). The extract was conserved at 4°C and protected from light and humidity.

### 2.3. Animals

One hundred *Meriones shawi* of both sexes were captured in the semiarid area of Boulmane in the Middle Atlas region of Morocco. Appropriate traps were used for catching the animals. They were then transported to the Faculty of Medicine and Pharmacy of Mohamed V University in Rabat, Morocco. Animals were allowed to adapt to the laboratory environment, numbered and placed in individual cages. All animals received a standard laboratory diet, *ad libitum*, which represents a hypercaloric food source for them. After three months of such diet, *Meriones shawi* having blood glucose greater than 8 mmol/L and having developed insulin resistance (AUC greater than 1500) were considered diabetic. This selection has enabled us to have a yield of 25% diabetic animals.

### 2.4. Experimental Procedure

Twenty-four diabetic and eight normal *Meriones shawi* were divided into four groups of 8 animals each as follows: normal control animals, diabetic control animals, diabetic NSE treated animals, and diabetic metformin-treated animals. Treatment was given by daily intragastric gavage at a dose 48 mg/kg/day of *N. sativa *extract (equivalent to 2 g plant/kg/day) and 300 mg/kg/day metformin for four weeks in one milliliter of 0.5% methyl cellulose suspension. Control animals received equal volume of vehicle (1 mL). Glycaemia and body weight were measured every week. At the end of the experimental period (4 weeks), the animals were fasted overnight, anaesthetized with an intraperitoneal injection of sodium pentobarbital (60 mg/kg), and sacrificed for obtaining blood and tissues samples (liver, soleus muscle). The study was conducted in accordance with the accepted principles outlined in the “Guide for the Care and Use of Laboratory Animals” prepared by the National Academy of Sciences and published by the National Institutes of Health and all efforts were made to minimize animal suffering and the number of animals used. Ethics approval was obtained from Mohammed V University.

### 2.5. Oral Glucose Tolerance Test (OGTT)

One day before the beginning and the end of the experiment, an Oral Glucose Tolerance Test (OGTT) was performed to assess glucose tolerance. For this purpose, overnight fasted rats were fed D-glucose (3 g/kg body weight) by intragastric gavage, and then blood was collected after 0, 15, 30, 60, and 120 min intervals from the tail vein. Plasma glucose concentrations were determined by the glucose oxydase method using a glucometer (One Touch Ultra, LifeScan Inc, Milpitas, CA). The areas under the curve (AUC) of changes in the blood glucose were calculated by using Origin software (Microcal Inc, Northampton, MA).

### 2.6. Plasma Lipid Assays

After 4 weeks of the treatment, *Meriones shawi* were sacrificed and fasting blood samples were collected for plasma chemical analysis. Total amount of cholesterol, LDL-cholesterol, HDL-cholesterol, serum triglyceride, and blood glucose were measured by an automated analyzer (Cobas-Mira Plus, Hoffman-LaRoche Diagnostics, Germany).

### 2.7. Radioimmunoassay

Plasma insulin, leptin, and adiponectin levels were determined by radioimmunoassay (RIA). Rat Insulin-specific RIA kit, Rat Leptin RIA kit, or Mouse Adiponectin RIA kit-125T were used. Generally, the samples were incubated in 12 × 75 mm polypropylene RIA tubes with rat ^125^I-insulin, ^125^I-leptin, or ^125^I-adiponectin and a primary antibody against rat insulin, rat leptin, or mouse adiponectin, respectively, at 4°C overnight in the dark. The tubes were then incubated with the precipitating reagent for 20 min at 4°C and centrifuged at 5350 g for 15 min. Radioactivity in the pellet was measured using a gamma counter (Wallac Wizard 1470, Perkin Elmer, Waltham, MA). Human insulin, rat leptin, or mouse adiponectin were used as respective standards.

### 2.8. Triglyceride Assay in Liver and Skeletal Muscle Tissues

Tissue was homogenized and extracted with a 2 : 1 chloroform-methanol mixture and washed by addition of 50 mM NaCl solution, resulting into two phases. The lower phase contained the total lipid extract. A fixed volume of this extract was dried, resuspended in isopropanol and an aliquot was used for triglyceride measurement using triglyceride and free glycerol reagents. Absorbance was measured at ambient temperature at 540 nm using a Wallac Victor 2 plate reader (Perkin-Elmer, Waltham, MA). Triglyceride content of the tissue was expressed as mg/g of wet weight of tissue.

### 2.9. Western Blot for Proteins Involved in Glucose Homeostasis

Samples of liver and skeletal muscle tissues were ground in liquid nitrogen and subsequently lysed. For ACC western blot analysis, 1 mL of RIPA lysis buffer was used (25 mM Tris-HCl pH 7.4, 25 mM NaCl, 0.5 mM EDTA, 1% Triton-X-100, 0.1% SDS), whereas sucrose lysis buffer (20 mM Tris-HCl pH 7.4, 255 mM sucrose, 1 mM EDTA) was used for Glut4. For all samples, a protease inhibitor cocktail was added (Roche, Mannheim, Germany) as well as 1 mM phenylmethanesulfonyl fluoride and phosphatase inhibitors (1 mM sodium orthovanadate, 10 mM sodium pyrophosphate, 10 mM sodium fluoride). Cells were allowed to lyse for 30 min on ice and were then centrifuged at 12000 × g for 10 min. Supernatants were then stored at −80°C until analysis. Protein content was assayed by the bicinchoninic acid method standardized to bovine serum albumin (Roche, Laval, QC). 

Lysates were diluted to a concentration of 1.25 mg/mL total protein and boiled for 5 min in reducing sample buffer (62.5 mM Tris-HCl pH 6.8, 2% SDS, 10% glycerol, 5%  *β*-mercaptoethanol and 0.01% bromophenol blue). 20 *μ*L of each sample was separated on 10% polyacrylamide mini-gels and transferred to nitrocellulose membrane (Millipore, Bedford, MA). Membranes were blocked for 2 h at room temperature with Tween-20 and 5% skim milk in TBS (20 mM Tris-HCl, pH 7.6 and 137 mM NaCl). Membranes were then incubated overnight at 4°C in blocking buffer with appropriate phospho-specific or pan-specific antibodies against ACC and Glut4 at 1 : 1000. Membranes were washed 5 times and incubated 1.5 h at room temperature in TBS plus Tween 20 with anti-rabbit HRP-conjugated secondary antibodies at 1 : 100000 to 1 : 50000. Revelation was performed using the enhanced chemiluminescence method and luminescence captured to blue-light-sensitive film (Amersham Biosciences, Buckinghanshire, England). Lysates from all experimental conditions were separated and transferred simultaneously to a single membrane.

### 2.10. Statistical Analysis

Data are reported as the mean ± SEM of the indicated number of experiments. Results were analysed by one-way analysis of variance (ANOVA) using StatView software (SAS Institute Inc, Cary, NC), with posthoc analysis as appropriate. Statistical significance was set at *P* ≤ .05.

## 3. Results

### 3.1. Body Weight Is Not Significantly Affected by NSE

The results of body weight are presented in Table 1. At the beginning of the trial period (day 0), the diabetic *Meriones shawi* weighed between 177 g–187 g as compared to approximately 158 g for non-diabetic controls. At the end of four weeks of treatment, an increment of weight of about 6 to 10 g for each *Meriones shawi* group was observed, but no significant difference was apparent between them, albeit the weight gain of NSE-treated animals had a tendency to be lower.

### 3.2. NSE Improves Blood Glucose of Diabetic *Meriones shawi*

**Figure 1:**
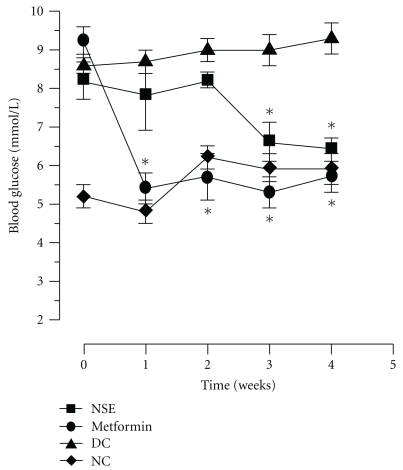
Changes of plasma glucose in diabetic *Meriones shawi* treated with NSE. *Meriones shawi* received a daily oral administration of NSE of 48 mg/kg or 300 mg/kg metformin (positive control) whereas normal control group (NC) and diabetic control (DC) groups received the methyl-cellulose vehicle. Results are expressed as mean ± SEM (*n* = 8). Significantly different from diabetic control (DC) group at the same time point, *P < .05.

Daily NSE treatment for four weeks resulted in a gradual decrease in glycemia that reached values similar to normal non-diabetic controls animals by the end of the treatment period (reduction from 8.2 ± 0.5 mmol/L to 6.4 ± 0.3 mmol/L, *P* < .05, *n* = 8, Figure 1). By comparison, metformin reduced blood glucose from 9.2 ± 0.4 mmol/L to 5.4 ± 0.4 mmol/L (*P* < .05, *n* = 8, Figure 1) within the first week and glycemia remained stable until the end of the study. In contrast, both diabetic and non-diabetic control animals displayed stable glycaemia during the four weeks of treatment; normal animals demonstrated normal blood sugar levels and control diabetic animals were hyperglycaemic throughout the study period (Figure 1).

### 3.3. NSE Reduces Insulin Resistance in the Diabetic *Meriones shawi*

**Figure 2:**
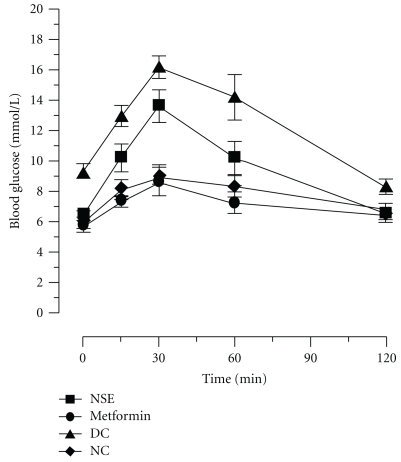
Effect of NSE on oral glucose tolerance test (OGTT) in diabetic *Meriones shawi*. OGTT (glucose 2 g/kg) was performed on fasted animals and blood glucose measured at the onset of glucose challenge (0 min) and at various time points afterwards (15–120 min). Results are expressed as mean ± SEM (*n* = 8). Significantly different from diabetic control (DC) *P < .05.

An oral glucose tolerance test (OGTT) was performed at the beginning of the study to identify the *Meriones shawi* that developed diabetes after three months of relatively hypercaloric diet. Another OGTT was also carried out on all selected study animals at the end of the treatment to determine the effect of NSE- and metformin-positive control on insulin resistance.  Figure 2 presents the changes in blood sugar observed during the two hours following administration of the glucose load. As can be seen, normal (non-diabetic) control animals had a small response to the glucose load as compared to control diabetic *Meriones*, indicating glucose intolerance/insulin resistance in the latter group. Treatment of diabetic animals for four weeks with the positive control metformin succeeded in completely restoring the OGTT response to that of non-diabetic congeners. In contrast, NSE-treated animals displayed a significant improvement in insulin sensitivity, but the glyceamic response to the OGTT was intermediate between normal and diabetic *Meriones* controls. The AUC of the glyceamic response to the glucose load over time confirmed and quantified the interpretation of Figure 2. Indeed, all diabetic animals showed similarly elevated AUC values at the beginning of the experimental protocol as compared to non-diabetic controls (Table 2). In diabetic *Meriones shawi* treated for four weeks with metformin, the values of the AUC fell significantly to reach values seen in non-diabetic controls. By comparison, NSE treatment for the same period significantly reduced the levels of AUC to values slightly greater than those of non-diabetic animals.

### 3.4. Plasma Lipid Profile: Differential Modulation by Metformin and NSE

In the diabetic *Meriones shawi*, treatment for 4 weeks with 48 mg/kg/day NSE or 300 mg/kg/day metformin increased total plasma cholesterol concentration by 49% and 38%, respectively, and the HDL-cholesterol concentration by 142% and 92%, respectively, as compared to the diabetic control group (Table 3). These increases brought levels of total cholesterol and HDL-cholesterol close to values observed in the normal control group, although HDL-cholesterol in NSE-treated animals had a tendency to be higher. Plasma LDL-cholesterol concentration was equivalent in all groups of *Meriones shawi* (Table 3), whereas metformin induced a significant rise in plasma triglycerides, as compared to control diabetic animals (Table 3).

### 3.5. NSE Increases Insulinemia but Does Not Affect Circulating Leptin or Adiponectin

**Figure 3:**
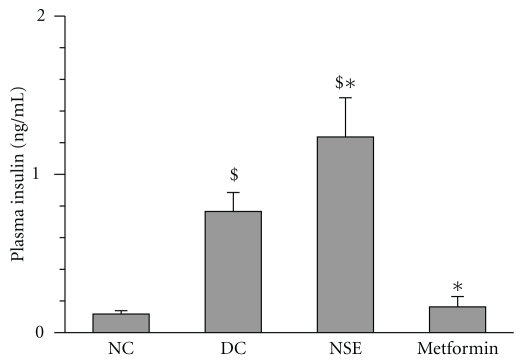
Effect of NSE on plasma insulin levels in *Meriones shawi*. The plasma insulin was measured after 4 weeks of treatment in diabetic control group (DC), normal control group (NC), and animals receiving 48 mg/kg/day *N. sativa* ethanolic extract (NSE) or 300 mg/kg/day of the reference oral antidiabetic drug metformin. Results are expressed as mean ± SEM (*n* = 8). Significantly different from diabetic control animals (DC) *P < 0.05. Significantly different from normal (non-diabetic) control animals (NC) ^$^ *P* < .05.

Untreated diabetic *Meriones shawi* exhibited a significant increase in plasma insulin as compared to their non-diabetic congeners (*P* < .05, *n* = 8, Figure 3). Treatment for four weeks with metformin reduced insulinemia back to levels observed in normal controls. In contrast, NSE treatment significantly increased plasma insulin levels beyond those observed in the diabetic control group (*P* < .05, *n* = 8, Figure 3).

The determination of plasma leptin in different groups of diabetic *Meriones shawi* yielded very similar values irrespective of NSE or metformin treatment. However, plasma leptin in normal *Meriones shawi* was found to be significantly higher than values observed in control diabetic animals (Table 3). In contrast, the results of plasma adiponectin showed no significant differences between groups, although NSE and metformin treated animals had a tendency to have lower adiponectin values (Table 3).

### 3.6. NSE Tends to Reduce Liver and Skeletal Muscle Triglyceride Content

As detailed in Table 3, liver and skeletal muscle triglyceride content appeared to be elevated in control diabetic versus non-diabetic *Meriones shawi* and metformin treatment did not change this tendency. In contrast, NSE had a tendency to decrease the triglyceride content of both tissues (Table 3). Because of the large data variability, however, none of these changes reached statistical significance.

### 3.7. NSE Increases Muscle ACC Phosphorylation and Glut4 Protein Content

**Figure 4:**
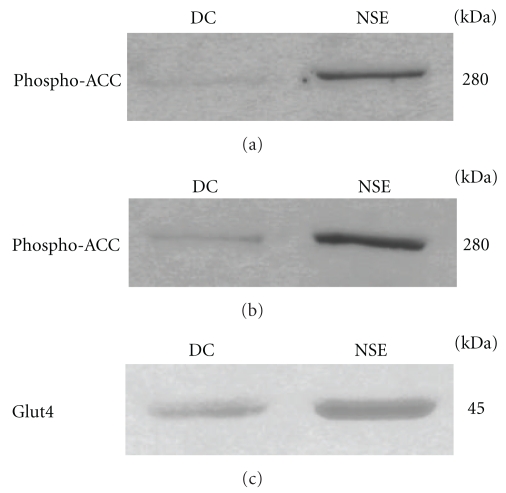
Effect of NSE on the phosphorylation of ACC in skeletal muscle and liver tissues and on Glut4 expression in skeletal muscle tissue. Samples of soleus muscle (a) and (c) and liver (b) tissues were obtained from diabetic *Meriones shawi* treated with NSE or vehicle (DC) and analysed by immunoblotting with antibody specific to phospho-ACC (a) and (b) and Glut4 (c). Immunoblots are representatives of results obtained from four animals of each group.

In order to begin understanding the mechanisms through which the effects of NSE are mediated, we assessed some key intracellular components involved in glucose homeostasis. We first evaluated the phosphorylation of ACC, a major component of the AMPK pathway. As shown in Figure 4, NSE treatment significantly increased the phosphorylation of ACC in liver (panel (a)) and skeletal muscle (panel (b)) in comparison with control diabetic* Meriones shawi*. Secondly, we probed the soleus muscle tissue of control and NSE-treated diabetic animals for their total content in Glut4 protein content. As illustrated in Figure 4(c), NSE substantially increased the amount of Glut4 present in the soleus muscle of diabetic *Meriones shawi* animals.

## 4. Discussion

**Figure 5:**
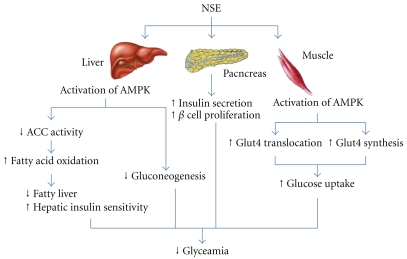
NSE activates AMPK in the liver and muscles to improve glucose metabolism. NSE mediates its action by stimulating the adenosine monophosphate-activated protein kinase (AMPK), an enzyme essential. It also reduces the enzymatic pathway involved in increasing fatty acid production by the liver (ACC = acteyl-CoA carboxylase); thus, it increases the sensitivity to insulin. The activation of AMPK can inhibit the pathway of gluconeogenesis in the liver. In the muscle, NSE activates AMPK towards allowing the increased synthesis and translocation of Glut4 and consequently increases glucose transport in muscle. NSE also acts on the pancreas by increasing the secretion of insulin.

*N. sativa* seeds are used in the traditional medicine of numerous Middle Eastern and North African countries for their antihyperglycemic activity [26–28]. Most studies to date have reported results from either normal animals or models of Type I diabetes [9, 12–15, 17–20], or from cells in culture [21, 24]. Only a single study presented limited evidence for an antidiabetic effect of *N. sativa* in an animal model of Type II diabetes [16]. The present study focussed on antidiabetic and hypolipidemic effects in the *Meriones shawi* model of Type II diabetes and aimed to elucidate the mechanisms of action of NSE in skeletal muscle and liver tissues (Figure 5).

*Meriones shawi* are rodents from semi-arid regions of Morocco that can gain weight and become insulin resistant and diabetic if kept in captivity (reduced physical activity) and fed normal laboratory chow (hypercaloric relative to their natural diet) [16]. This was confirmed in our study by the greater body weight of the *Meriones shawi* animals that also demonstrated insulin resistance as assessed by an OGTT, as compared to non-diabetic animals maintaining normal glucose tolerance. Treatment with NSE or metformin for four weeks failed to significantly affect body weight, although a tendency for smaller weight gain was seen in NSE-treated animals as compared to untreated diabetic controls. This contrasts with the results of Labhal and collaborators who reported a decrease in body weight among *Meriones shawi* treated with an aqueous extract of *N. sativa *[16]; similar results having been obtained with the sand rat *Psammomys obesus *[8]. Aside from the difference in *N. sativa* extract type, the treatment period of these studies lasted three months. It is therefore possible that our shorter treatment regimen and use of an ethanol extract may explain the lack of an effect on body weight in the present studies.

In contrast, our studies confirmed the oral hypoglycemic action of *N. sativa* in an animal model of Type II diabetes. As mentioned, such a decrease in blood glucose was reported in several studies using animal models of type I [9, 10, 15, 17–20] and on similar models of Type II diabetes [8, 16]. In our experimental conditions, NSE decreased blood sugar starting from the 3rd week of treatment whereas metformin normalized glycaemia within first week. It is known that thiazolidinediones (TZD) also decrease blood sugar after 3 or 4 weeks of treatment [29]. *In vitro* studies in our laboratory have confirmed that NSE stimulates PPAR-*γ* in cultured adipocytes as do TZD [25]. Part of the action of NSE on the regulation of blood glucose *in vivo* may therefore be similar to TZDs. However, the lack of effect of NSE on plasma leptin and adiponectin indicates that the action of NSE on adipose tissue may not implicate the modulation of these two adipokines in the *Meriones shawi* model.

Moreover, the decrease of blood glucose by NSE was associated with a significant reduction in insulin resistance, as revealed by the pattern of the OGTT response in diabetic animals and the resulting significant decrease in AUC that clearly indicate an improvement in glucose tolerance. This improvement in insulin sensitivity is fully consistent with our previous results showing increases of basal and insulin stimulated phospho-Akt in hepatocytes isolated from normal rats treated with the petroleum ether extract of *N. sativa *[22].

NSE treatment also modulated the lipid profile of diabetic *Meriones shawi*, most notably by significantly increasing HDL-cholesterol, an effect that we have previously observed in normal rats with a different *N. sativa* extract [22]. We also observed a tendency towards a decrease in triglycerides in the NSE-treated group.  Labhal et al. [16] observed a significant decrease in blood triglycerides in the same animal model using an aqueous extract of *N. sativa* administered for a period of three months. Once again, differences in extract type and treatment period may explain discrepancies with our results at the level of blood lipid profile. 

However, our studies went further by assessing triglyceride content in insulin-sensitive tissues, notably skeletal muscle and liver. Although the apparent NSE induced decrease in these parameters failed to reach statistical significance, such an action could participate in the improvement of systemic insulin sensitivity observed in our studies. Indeed, it is known that high levels of intracellular triglycerides can increase some lipid metabolites such as ceramides, diacylglycerol, and long-chain acyl-coenzyme A [30]. The latter play a key role in attenuating insulin signaling by increasing intracellular serine-threonine phosphorylation of IRS protein, with a resultant reduction in insulin signal transduction that underlies insulin resistance [30].

More importantly, the NSE-treated *Meriones shawi* also showed a large increase in insulinemia after 4 weeks of treatment that can contribute to the antihyperglycemic effect of NSE. Such an increase was previously observed by our group in normal rats [22] and is also observed in streptozotocin-nicotinamide hamsters [18]. In addition, treatment of pancreatic *β* cells in culture with *N. sativa* was found to increase insulin secretion as a result of an improved secretagogue capacity of these cells [21]. On the other hand, *N. sativa* increases regeneration of pancreatic *β* cells [20] and protects the same from streptozotocin [10]. Our own recent *in vitro* studies clearly demonstrate that NSE can enhance the proliferation of *β* cells and increase glucose stimulated insulin secretion [24]. Taken together, these actions can explain the important increase in insulin levels observed in diabetic NSE-treated* Meriones shawi* (Figure 5). They further support the notion that *N. sativa* products can help maintain pancreatic *β*-cell mass and hence mitigate the progression of diabetes. Future studies will have to ascertain that this also occurs in the *Merione shawi* model.

Our studies also attempted to examine certain key intracellular components involved in glucose homeostasis. Western blot analysis showed that NSE treatment *in vivo* can significantly increase the total amount of Glut4 glucose transporters in skeletal muscle, which play a major role in controlling hyperglycemia [31]. Moreover, Glut4 proteins are known to be subject to transcriptional regulation that allows for their increased synthesis [32], with a resultant contribution in reducing hyperglycemia. Further studies will be necessary to determine the mechanisms underlying the action of NSE to increase skeletal muscle Glut4, but this action is likely very relevant to the overall glucose-lowering activity of the plant (Figure 5). 

Finally, we assessed ACC, a key component of the insulin-independent, metabolic sensing, AMPK pathway [33]. Indeed, our group recently reported on the stimulation of ACC and the AMPK pathway by NSE in both skeletal muscle and hepatocyte cell lines *in vitro *[25]. In the present studies, we found that NSE treatment *in vivo* can increase the phosphorylation of ACC in liver and skeletal muscle tissues. The phosphorylation of ACC reduces its activity and results in a decrease of lipogenesis in the liver and an increase of fatty acid oxidation in skeletal muscle [34]. These effects on lipid metabolism can underlie the tendency for NSE to reduce plasma and tissue triglycerides observed herein. Lastly, it is known that the activation of the AMPK pathway can lead to increased synthesis of Glut4 [35], and this is also in accordance with our results (Figure 5).

In conclusion, NSE greatly improves systemic glucose homeostasis and HDL-cholesterol in diabetic *Meriones shawi* by acting through several mechanisms. Most importantly, *N. sativa* increases circulating insulin and enhances the sensitivity of peripheral tissues to the hormone. The latter effect can be attributed in part to an activation of the AMPK pathway in skeletal muscle and liver and to an increased content of Glut4 in skeletal muscle (Figure 5). Such pleiotropic actions provide strong evidence in support of the traditional use of *N. sativa* seeds for the treatment of diabetes. They further call for high-quality clinical studies to determine the optimal conditions for complementary or alternative treatment in diabetic patients.

## Figures and Tables

**Table 1:** Evolution of body weight over the course of the 4-week treatment.

Time (week)	*N. sativa* (g ± SEM)	Metformin (g ± SEM)	Diabetic control (g ± SEM)	Normal control (g ± SEM)
0	178 ± 17	177 ± 11	187 ± 15	158 ± 14
1	172 ± 16	166 ± 11	178 ± 15	153 ± 12
2	183 ± 19	180 ± 10	186 ± 15	161 ± 13
3	185 ± 18	183 ± 9	192 ± 16	166 ± 13
4	184 ± 16	184 ± 9	197 ± 15	170 ± 12

No statistically significant differences were observed between experimental groups at any of the time points (*n* = 8 per group, N.S.).

**Table 2:** Area-under-the-curve (glyceamic (mmol/L)*time (min)) for OGTT before and after 4-week treatment.

Time (week)	Treatment			
	*N. sativa*	Metformin	Diabetic control	Normal control
0	1648 ± 107	1685 ± 84	1666 ± 82	956 ± 61^§^
4	1163 ± 81^∗§^	819 ± 54^∗§^	1514 ± 83	941 ± 41^§^

*Significantly different from week 0; *P* < .05; ^§^Significantly different from Diabetic controls; *P* < .05.

**Table 3:** Effect of NSE on blood biochemistry and tissue lipid parameters.

	Normal control	Diabetic control	*N. sativa*	Metformin
Plasma total cholesterol (mmol/L)	0.86 ± 0.09	0.65 ± 0.08	0.97 ± 0.08*	0.90 ± 0.10
Plasma LDL-cholesterol (mmol/L)	0.13 ± 0.04	0.10 ± 0.06	0.16 ± 0.05	0.08 ± 0.06
Plasma HDL-cholesterol (mmol/L)	0.48 ± 0.07	0.26 ± 0.05	0.63 ± 0.04*	0.50 ± 0.08*
Plasma triglyceride (mmol/L)	0.55 ± 0.08	0.50 ± 0.04	0.41 ± 0.08	0.72 ± 0.09*
Plasma leptin (ng/mL)	8.60 ± 2.00	3.50 ± 1.20	3.20 ± 0.50	5.00 ± 1.70
plasma adiponectin (*μ*g/mL)	8.59 ± 1.86	8.50 ± 1.34	5.51 ± 1.32	6.35 ± 1.86
Liver triglyceride (mg/g of tissue)	35.1 ± 18.0	83.3 ± 43.2	28.1 ± 13.5	79.8 ± 31.6
Muscle triglyceride (mg/g of tissue)	5.23 ± 1.10	8.92 ± 3.50	4.23 ± 0.78	10.5 ± 4.39

*Significantly different from Diabetic controls; *P* < .05.
